# High impact: Examining predictors of faculty-undergraduate coauthored publication and presentation in psychology

**DOI:** 10.1371/journal.pone.0265074

**Published:** 2022-03-31

**Authors:** Traci A. Giuliano, Isham E. Kimbell, Emily S. Olson, Jennifer L. Howell

**Affiliations:** 1 Department of Psychology, Southwestern University, Georgetown, Texas, United States of America; 2 Department of Psychological Sciences, University of California Merced, Merced, California, United States of America; Universitat de Valencia, SPAIN

## Abstract

Despite the increasing popularity of faculty-undergraduate research, a dearth of research has investigated factors that predict the professional outcomes of these collaborations. We sought to address this gap by examining a wide range of institutional (e.g., institution type, selectivity, course load) and faculty variables (e.g., rank, years of experience, enjoyment of mentoring) potentially related to coauthored undergraduate publication and conference presentation in psychology. Negative binomial regressions were used to analyze online survey data from 244 faculty members from both graduate-serving institutions (i.e., doctoral, master’s) and primarily undergraduate institutions. The results showed that, after controlling for overall research productivity, faculty at primarily undergraduate institutions were more likely to publish journal articles with undergraduates, whereas faculty at graduate-serving institutions were more likely to coauthor conference presentations with undergraduates. Institutions with higher selectivity, more support for faculty-undergraduate research, and lower course loads produced higher numbers of undergraduate publications. Faculty characteristics were even more strongly related to undergraduate research outcomes. Specifically, publication was most likely with faculty who are of higher rank, have more years of experience, spend more time on research, foster close collaborative relationships with undergraduates, and/or perceive their students as high quality and well trained. By contrast, conference presentation was most likely with faculty who work with more undergraduate students on more projects per year and/or who enjoying mentoring undergraduates. Our findings suggest ways that institutions can facilitate undergraduate publication, which we argue is an increasingly common and achievable outcome.

## Introduction

Colleges and universities are increasingly emphasizing the engagement of undergraduate students in “high-impact” experiences, particularly faculty-student research collaboration [1–5]. Such experiences provide benefits for all involved. Students gain research, problem-solving, and communication skills and are favorably positioned for graduate school and other careers [6]; faculty gain satisfaction and the advancement of their teaching and research goals [6, 7]; and institutions strengthen their educational quality and improve recruitment and retention [1, 8–10]. Unfortunately, despite extensive documentation of the benefits of faculty-undergraduate research collaboration [10, Chapter 3], and some studies examining predictors of mentoring undergraduates in research [6, 10], relatively little is known about the outcomes of such collaborations nor the factors that facilitate successful outcomes. Due to the increasing interest and enthusiasm for publishing with undergraduates in psychology (e.g., [11]), and because the few empirical studies of undergraduate publication have thus far only focused on biomedical sciences [12–14], we sought to examine the frequency and predictors of two research outcomes in psychology: faculty-undergraduate coauthored publication in peer-reviewed journals and faculty-undergraduate coauthored conference presentations.

### Facilitators of, and barriers to, faculty-undergraduate research

Given that faculty-undergraduate research collaboration can be beneficial to everyone involved, it is important to understand factors that can facilitate or hinder such collaboration. We consider here two broad categories of factors: Institutional factors (i.e., those outside the faculty member, such as course loads and institutional support) and individual faculty factors (i.e., those within the faculty member, such as their background, attitudes, and experiences).

**Institutional factors.** At the institutional level, direct support for undergraduate research (e.g., in the form of time and money to conduct the research as well as rewards and recognition for faculty who mentor undergraduates) facilitates faculty-undergraduate research collaboration. Otherwise, financial constraints can be a challenge, with faculty citing both a lack of money for lab supplies [7, 15], and the need to personally find ways to fund students [6] as barriers to mentoring undergraduates in research.

Another institutional factor that increases the likelihood of mentoring undergraduates is having undergraduate research incorporated into faculty course load, which helps faculty maximize their time by combining teaching and research [7]. Consistent with this notion, overall workload has been shown to adversely affect faculty interest in mentoring undergraduate research [16, 17] and to make the development of high-quality mentoring relationships more difficult when faculty do decide to collaborate [16].

Finally, institutional support in the form of recognition by the administration can be critical [18]. Unfortunately, faculty often report that research is encouraged and ostensibly valued, but not actually built into the formal reward structure of the university [7, 15, 19, 20]. A common complaint is that “My institution ‘values’ undergraduate research, but in terms of annual evals and promotion and tenure, it is not really given any weight” [6, p. 145]. Predictably, faculty who perceive that mentoring undergraduates is not rewarded by their university are less interested in devoting their limited time and energy toward mentoring such research [21].

#### Faculty factors

At the individual faculty member level, factors that predict mentoring undergraduate research take many forms—from the availability of time and money to perceptions of student quality to career stage. Not surprisingly, the most commonly mentioned barrier by faculty is time [7, 15, 22–29]. Mentoring undergraduates takes a lot of effort because they require more training, supervision, and one-on-one time than would a typical graduate student [30]. Indeed, studies have found that especially for new or untenured faculty, who are busy preparing classes, doing administrative work, and establishing a research program, the return on investment may not seem worthwhile given the amount of time it takes to mentor undergraduate research [6]. In a similar vein, faculty often experience a lot of pressure to publish in high-impact journals—and to do so rapidly [31]—and thus they may feel that undergraduate projects are a barrier to this end goal. In addition to time, money is needed for faculty-undergraduate research to cover expenses from materials and software to participant compensation to publication fees. Thus, it is not surprising that faculty who have received external grants are not only more likely to participate in structured undergraduate research programs [10] but also more likely to involve undergraduates in their research overall [14, 32, 33].

Yet another barrier (which may be percieved rather than actual [6]) is a lack of interested, qualified students. A recent study of 120 members of the American Statistical Association found that 61% cited a lack of students interested in undergraduate research or willing to take the time necessary to do undergraduate research; 31% of faculty in that study also did not believe that their curriculum was robust enough to prepare students for meaningful statistics research [6]. Echoing these results, 35% of another sample of faculty felt that undergraduates could not make significant contributions to their research [15], although this belief was much more prevalent among faculty who had not previously collaborated with undergraduates (28%) than in those who had (7%).

Finally, faculty career stage can be a factor: Potter et al. [33] found that full professors (relative to assistant and associate professors) reported more satisfaction mentoring undergraduates. Indeed, the full-professor rank may be the ideal stage to mentor undergraduates because a faculty member’s research program is already well-established, and more extensive experience makes them better suited to effectively serve as mentors [33].

### Predictors of faculty-undergraduate research outcomes

Despite the wealth of empirical research on factors that predict faculty engagement in research collaboration with undergraduates (e.g., [6, 15]), little is known about the actual outcomes of these collaborations. Perhaps because undergraduate authors are not easily identifiable on faculty publications, it is difficult to determine the frequency with which undergraduates coauthor journal articles. The dearth of base rates and literature in this area also makes it difficult to make predictions about the factors that lead to undergraduate publication.

Although there are no empirical studies on undergraduate publication in psychology, there are a few such studies in other fields that can possibly offer insight. Mellis et al. [13] examined data from 4 PUIs over a 10-year period from 2004 to 2013 (including 59 faculty and 548 research students) to identify factors that determine undergraduate presentation and publication in chemistry and physics. They found that although the majority of the outcomes were conference presentations (85%), a significant number (15%) were peer-reviewed publications. Furthermore, faculty rank was a significant factor (i.e., higher rank was related to more undergraduate publications) in chemistry but not in physics. In a similar study but using research-intensive institutions, Morales et al. [14] surveyed 468 biomedical sciences faculty at 13 R1 (i.e., doctoral universities with very high research activity) programs and found that years working in higher education, external grant funding, enjoyment of mentoring undergraduate research, prior experience mentoring African-American students, and length of the research experience (i.e., working with undergraduates for longer than a year) were all related to publishing more with undergraduates. Without comparable data in psychology, it is difficult to determine the extent to which these findings generalize to our field.

### The current study

Taken together, the literature indicates that despite the potential barriers for faculty wishing to mentor undergraduate research, there are strategies to overcome such barriers. Importantly, faculty-undergraduate research is on the rise in recent years [4], which is fortunate given the many well-documented benefits for students, faculty, and institutions [6]. However, there is a dearth of research on the actual outcomes of faculty-undergraduate research in psychology, including the frequency with which undergraduates coauthor peer-reviewed journal articles and the factors that predict faculty-undergraduate publication. In the present study, we sought to address this gap by surveying psychology faculty members at a variety of U.S. colleges and universities to investigate predictors of publishing with undergraduate students. We examined two primary questions: Which institutional factors (e.g., institution type, selectivity, support for undergraduate research, course load) and which faculty characteristics (e.g., rank, experience, attitudes about mentoring undergraduates, area of psychology) predict the likelihood of publishing with undergraduates? We also examined whether these same factors predicted publishing with undergraduates as first author, conference presentations with undergraduate coauthors, and conference presentations with undergraduates as first author.

## Method

### Participants and procedure

In general, our goal was to obtain the largest convenience sample possible while staying within budget constraints (the first author had $1500 in departmental research funds that we could allocate to 150 participants in the form of $10 Amazon gift cards). Our power analysis (α = .05, 1-B = .80, and assuming a small correlation of .21 [34]) indicated a minimum required sample size of 138. We thought it was important to include a variety of institution types to examine the ways in which faculty-undergraduate publication might differ at institutions that had undergraduates vs. graduate students, varied expectations for teaching and scholarship, and differences in faculty course load. Thus, we targeted three institution types based on the Carnegie Classification of Institutions of Higher Education [35]. From their classification system, we chose doctoral universities with very high research activity (R1), master’s universities (M1, M2, and M3), and baccalaureate colleges with an arts and sciences focus. Because we were interested in using university rank as a variable (i.e., as a potential predictor of publishing with undergraduates), we used the U.S. News and World Report [36] rankings for our original sampling frame which comprised a list of the top 100 institutions of each type.

Given the focus of our study, we targeted faculty who had significant experience conducting research with undergraduates. In early March of 2020, we emailed department chairs of the 300 departments on our list and asked them to identify the person in their department who did the most collaborative research with undergraduates. For departments whose chairs failed to respond, we searched department web pages to identify a faculty member who appeared to conduct research with undergraduates. Links to the online survey were sent to faculty in late May and early June of 2020, and non-responders received three reminder emails approximately 4 to 7 days apart. This research was approved by the Southwestern University Institutional Review Board, and participants gave written consent before starting the online survey.

Of the 300 faculty members contacted, 72 completed surveys (which represented a 20% response rate). To increase our sample size, we next contacted faculty who had shown interest in contributing to a special journal issue on the topic of publishing with undergraduates [11]. These 196 solicitations resulted in 114 completed surveys. Once funds were exhausted, we made public appeals for voluntary participation through announcements on social media, which yielded 58 additional surveys. The final sample included 244 faculty participants (45 from R1 universities, 64 from master’s universities, 119 from national liberal arts colleges, and 15 from some other type of institution, such as R2s or regional campuses of R1s). Throughout the paper, when referring to school type, we focus mainly on the distinction between primarily undergraduate institutions (PUIs; 53.9%) and graduate-serving institutions (GSIs; 46.1%). Full sample demographics and responses on measures appear in Table 1.

**Table 1:** List of variables, descriptive statistics, predicted relationship with number of undergraduate (UG) publications, and associated citations^1^.

Variable	M (SD) or %	Expected Direction of Relationship	Relevant Citation
**Institution**			
** Institution Type**		Positive	(33)
PUI (Primarily Undergraduate)	53.9%		
Grad-Serving (Masters or Doctoral)	45.9%		
Rank Within Type (Lower = Better Rank)	64.84 (65.46)	Negative	(37)
Endowment in Millions^2^	511.61 (1149.07)	Positive	(10)
Acceptance Rate (Higher = Less Selective)	63.0% (21.0%)	Negative	
Student:Faculty Ratio	13.06: 1 (4.14)	Exploratory	
Support for Undergraduate Research	3.68 (0.97)	Positive	(6,18)
**Department**			
# of Faculty	14.42 (13.74)	Exploratory	
Undergraduate Research in Curriculum	3.77 (1.16)	Positive	(7)
Undergraduate Research in Course Load	41.8%	Positive	(38)
Teaching Load	5.46 (2.47)	Negative	(7,18)
**Faculty Characteristics**			
**Ethnicity**^3^		Not included	
White, non-Hispanic	91.80%		
Hispanic or Latinx	1.60%		
Black or African American	0.80%		
Asian American	2.90%		
Multi-Racial	2.50%		
Other	0.40%		
Age	45.95 (10.99)	Positive	(14)
**Gender**^4^		Exploratory	
Female	67.6%		
Male	31.8%		
Non-binary	0.8%		
Years as a Full-Time Faculty Member^7^	15.90 (10.84)	Positive	(14)
**Tenure Track Rank**^5^		Positive	
Assistant	18.8%		(14,33)
Associate	36.0%		
Full	45.2%		
Grant Recipient (> $5k)	35.4%	Positive	(1,14)
# of Publications^8^	21.81 (36.29)	Positive	(33)
Hours Worked Per Week	49.86 (9.99)	Positive	
Hours Spent on Research	14.09 (8.96)	Positive	
Hours Spent Teaching Per Course	5.22 (2.40)	Negative	(15,32)
Satisfaction with Current Position	4.04 (0.93)	Positive	
**Research Lab**			
# Grad Students in Lab^6^	1.29 (2.70)	Exploratory	
# Undergrads in Lab	8.78 (9.79)	Positive	(14)
Experience with Diverse Students	4.01 (0.83)	Positive	(10)
** Collaboration**			
% of RAs Who Are Juniors and Seniors	80.51 (17.86)	Exploratory	
% of RAs Who Are Primarily "Assistants"	35.27 (31.71)	Exploratory	
% of RAs Who Are Primarily "Collaborators"	56.91 (33.88)	Exploratory	
Length of Collaboration with Undergrads	12.92 (7.33)	Exploratory	
** Collaborations with other Faculty**			
Primarily Students (no outside faculty)	3.00 (1.33)	Exploratory	
Within Department	2.60 (1.33)	Exploratory	
Outside Department but Within University	2.12 (1.27)	Exploratory	
Outside the University	3.38 (1.45)	Exploratory	
** Collaborations with Students**			
Primarily Work Alone on Projects (no students)	2.01 (1.05)	Exploratory	
Primarily Undergraduate Lab	3.19 (1.34)	Positive	(14)
Both Grad and Undergrad Co-Investigators^6^	1.87 (1.44)	Exploratory	
Primarily Grad, Expected Mentoring of UG^6^	1.52 (1.08)	Exploratory	
PI Supervises Grad Who Supervises UG^6^	1.24 (0.62)	Exploratory	
** Faculty-Student Interaction Styles**			
Collegial Relationships with Undergraduates	4.52 (0.66)	Positive	(39,40)
Undergraduates Impact Project Direction	4.03 (0.91)	Positive	
Very Accessible to Undergraduates	4.51 (0.66)	Positive	(23)
Clear Expectations for Undergraduates	4.22 (0.67)	Positive	(11)
**Project Characteristics**			
Original Projects (v. Replications)	4.50 (0.71)	Exploratory	
Number of Projects Per Year	3.78 (3.17)	Exploratory	
Study Length (in Minutes)^9^	49.35 (52.93)	Exploratory	
** Participants**			
** Primary participants/subjects**			
Animals	7.8%	Exploratory	
Adults	83.2%	Exploratory	
Children	9.0%		
Average number of Participants Per Study	157.82 (190.13)	Exploratory	
** Data Collection**			
Primarily Online	69.30%	Exploratory	
Primarily With Individuals in Person	68.40%	Exploratory	
Primarily With Groups in Person	30.70%	Exploratory	
**Faculty Perceptions of Students & Research**			
Student Quality	3.54 (0.97)	Positive	(10,14)(15)
Benefit (vs. Cost) of Research With UG	3.08 (1.03)	Positive	
Enjoyment of Collaborating With UG	4.56 (0.51)	Positive	
Perceived Benefit to UG (3-item index) of conducting/presenting/publishing research	4.78 (0.35)	Positive	

1 = See pre-registration for more detail

Regression analyses

2 = Excluded one university who had 4x the endowment as the next closest school (≥ 12 SD above the mean)

3 = Did not include ethnicity because the sample was 92% White

4 = Focused on men and women (99% of the sample)

5 = Focused on tenure-track professors (98% of the sample)

6 = For these variables, included faculty at graduate-serving institutions only

7 = We recoded one faculty response from “3232 years” to “32 years.”

8 = Four faculty reported extremely high numbers of publications with undergraduates (43, 50, 68, and 68) more than 3 SD above the mean and median and were thus removed for analyses of number of publications with undergraduates. Their data were still retained for all other outcomes.

9 = Examined only studies under 400 minutes (6.67 hours), excluding one faculty member who indicated that participation in their studies takes 100 24-hour days, and two faculty who indicated that their studies took 16.67 and 20 hours, respectively (all ≥ 17 SD above the mean).

### Measures

The primary criterion variables were outcomes of undergraduate research collaboration, including peer-reviewed journal publications and conference presentations, both in general and as first author. Although publications, and in particular first-authored publications, perhaps represent the gold standard outcome of research in psychology, we included conference presentations as well because they represent a potentially important outcome of undergraduate research with a lower barrier to entry. Predictor variables were grouped into six categories: institutional variables, department variables, faculty characteristics, faculty perceptions, research lab variables, and research project characteristics. A list of variables and descriptive statistics for each appear in Table 1. The preregistration of hypotheses, analysis plan, and data can be found at https://osf.io/87em9/. We encourage readers interested in questions we do not address here to download and explore the data.

Below we detail our primary constructs. A full list of constructs and descriptive statistics for each appears in Table 1. Exact wording of all self-report items appears in Table 2.

**Table 2:** Wording of self-report items for primary variables (UG = Undergraduate).

Construct	Wording of Items
Institutional Support for Mentoring UGs	• My institution encourages/supports/rewards faculty for conducting research with undergraduates • My institution encourages/supports/rewards faculty for publishing research with undergraduates
Teaching Load	• Number of courses taught per year, on average
UG Research in Teaching Load	• Is conducting research with undergraduates part of your course load (i.e., incorporated into your teaching load/counted as a class)?
Curric. Structured to Foster UG Research	• My department’s curriculum is carefully structured/scaffolded to build undergraduates’ research skills • Many of our undergraduate courses incorporate opportunities to conduct original research as part of the course
Job Satisfaction	• Overall, how satisfied are you in your current academic position?
Perception of Student Quality	• It is easy to find high quality undergraduate research assistants at my institution • Undergraduates at my institution are often not equipped to make significant contributions to my research area
Cost-Benefit Ratio of Mentoring UGs	• Conducting research with undergraduates is more time consuming than worthwhile • Doing research with undergraduates sometimes has more costs than benefits
Enjoyment/Commitment to UG research	• I truly enjoy training undergraduates to do research • Mentoring undergraduates through the research process is an important part of my job • My past experience working with undergraduates has been very positive
Perceived Benefits of Research to UGs	• I believe that conducting empirical research has substantial benefits for undergraduates • I believe that presenting conference papers/posters has substantial benefits for undergraduates • I believe that publishing/coauthoring peer-reviewed papers has substantial benefits for undergraduates
Past Experience with Diverse UGs	• I have experience collaborating with undergraduate research assistants from diverse racial/ethnic backgrounds • I go out of my way to seek/select undergraduate research assistants from diverse racial/ethnic backgrounds
Collaboration Model in Faculty Research Lab	• I generally work on my own projects with very little involvement from grad students or undergrads • I work on my projects exclusively with grad students; they recruit undergrads to help with minor lab tasks when needed (e.g., running participants, data entry) but I have little to no contact with the undergraduates • I work on my projects primarily with graduate students who I expect to thoroughly mentor undergraduates in our lab through our research process • I work on my projects with both graduate and undergraduate students and all of them are invited to participate and contribute to the project at all stages • I work on my projects exclusively with undergraduate students who are full, equal collaborators through all stages of the research process
Faculty Mentoring Style with UGs	• I try to establish close, collegial relationships with my undergraduate research assistants • I allow my undergrads to have significant impact on the topic/direction of our research • I am very accessible to my undergrad research assistants • I provide very clear expectations for my undergrad research assistants
Collaboration with Other Colleagues	• I tend to work primarily with my students and don’t collaborate much with other faculty • I tend to collaborate with colleagues in my psychology department • I tend to collaborate with colleagues outside of my department but within the university • I tend to collaborate with psychology colleagues at other institutions
Type of Research	• Which best matches your research with undergraduates? 1 = *Primarily Replication*, 2 = *More Replication than Original*, 3 = *Equal Replication/Original*, 4 = *More Original than Replication*, 5 = *Primarily Original*”

#### Institutional variables

At the beginning of the survey, we asked participants to classify their institution type as *national doctoral/R1*, *regional/masters (M1/M2/M3*), *national liberal arts (baccalaureate; small liberal arts college/SLAC)*, or *other*. We used these responses to categorize researchers as being at either (1) primarily undergraduate institutions (PUIs; institutions that do not have graduate students) or (2) graduate-serving institutions (GSIs; institutions that do have graduate students).

We then asked the extent to which faculty perceived institutional support for mentoring undergraduate research using two items (1 = *Strongly Disagree*, 5 = *Strongly Agree*), *r*(244) = .66, *p* < .001. (See Table 2 for the wording of all self-report items measured).

At the end of the survey, participants were given the option of listing the name of their institution; 74% (181/244) of participants opted to do so. We used these names to gather additional data on the institutions’ rank within institution type (from the U.S. News and World Report 2021 rankings); we further retrieved university information (i.e., endowment, student acceptance rate or selectivity, and student-faculty ratio) from university webpages in February of 2021.

#### Department variables

First, we asked faculty the number of full-time tenure-track faculty in their department. We then assessed their teaching load and asked whether undergraduate research was part of their course load. Finally, we measured whether their curriculum was structured to foster undergraduate research, using two items (1 = *Strongly Disagree*, 5 = *Strongly Agree*), *r*(43) = .62, *p* < .001.

#### Faculty characteristics

Background questions used in predictive analyses assessed participants’ age, gender, years at current institution, rank (*assistant professor*, *associate professor*, *full professor*, *visiting/adjunct/non-tenure-track*), and years as a full-time faculty member in higher education. We also asked participants to identify their research area/subfield (*animal behavior*, *clinical*, *cognitive*, *developmental*, *evolutionary*, *general experimental*, *I/O*, *neuroscience*, *personality*, *social*, and *other*). We then asked whether participants had received an external grant as a P.I. greater than $5000 in the last 10 years and about their overall research productivity (number of peer-reviewed journal articles as a professor).

Next, we measured faculty workload. We asked the number of total hours worked per week on academic tasks and the relative percent of time spent on teaching, research, and service. We used these metrics to compute the total number of hours faculty spent on research (hours per week x percentage time spent on research), and hours spent teaching each course (hours per week x percentage of time spent on teaching divided by course load). Finally, we assessed faculty job satisfaction (1 = *Not at All Satisfied*; 5 = *Very Satisfied*).

#### Faculty perceptions of students and research

After measuring faculty characteristics, we used several Likert-scale items (1 = *Strongly Disagree*, 5 = *Strongly Agree*) to measure faculty attitudes toward undergraduate research. Two items measured perceptions of student quality [*r*(243) = -.53, *p* < .001] and two items assessed the perceived cost-benefit ratio of mentoring undergraduates [*r*(243) = .66, *p* < .001]. An additional 3-item index (α = .74) assessed enjoyment of, and commitment to, research collaboration with undergraduates. Finally, a 3-item index (α = .66) assessed perceptions of the benefits of research to undergraduates.

#### Research lab variables

To determine typical laboratory composition, we measured the number of graduate students and undergraduate students worked with in an average year. Two additional items assessed past experience collaborating with diverse undergraduates, *r*(243) = .52, *p* < .001. We then asked faculty to indicate the percentage of undergraduates they worked with who were first-years, sophomores, juniors, and seniors. We recoded this as a dichotomous variable so that it represented the percentage of undergraduate RAs who were juniors and seniors (compared to first-years or sophomores).

To assess the collaboration model in a faculty member’s research lab, we asked faculty to describe the extent to which each of five statements (analyzed individually) characterized their research lab (1 = *Not at All True*, 5 = *Very True*; e.g., as having little involvement from graduate or undergraduate students, high involvement of both groups, or involvement with one group but not the other). For the variables referring to graduate students, we restricted analyses to participants at graduate-serving institutions only.

We then sought to capture the extent to which a faculty member’s undergraduate mentoring style was close and collaborative with four questions analyzed individually. Similarly, we asked faculty to describe the percent of undergraduate research assistants in a typical year who play the role of “assistants” (i.e., collecting data but not helping with literature review, study design, analysis, and writeup), “collaborators” (i.e., involved in all stages of the research process, including literature, study design, data collection, analysis, and write-up), or “other.” The total added up to more than 100% for multiple faculty participants, so we focused on percentage of “assistants” and “collaborators” as separate predictor variables. We also assessed the average duration (in months) of collaboration with the typical undergraduate.

Next, we measured collaboration with other faculty. Using four items analyzed individually, we asked participants to indicate the extent that their research (1 = *Not at All True*, 5 = *Very True*) involved collaborating with other colleagues within or outside the university.

#### Research project characteristics

To investigate project characteristics, we first asked faculty how much they conducted original vs. replication research with undergraduates. Next, we asked about the number of different projects in a typical year that undergraduates would be working on. We also assessed average number of participants and average length of time required of participants in a typical study. Faculty then indicated whether they typically collect data *with individuals in person*, *with groups in person*, *online*, or *other* and their most common participant (*Child/Under 18*, *College Student*, *Adult/Non-Student/Community Member*, or *Animals*). We recoded these variables that examined typical research participants into a series of Helmert contrasts that compared people who studied animals (.67) vs. humans (children or adults = -.33), and then into people who studied children (.5) vs. adults (-.5; animals = 0).

#### Outcomes of undergraduate research

Our primary criterion measures were the extent to which faculty had (1) published coauthored journal articles and (2) presented at conferences with undergraduate students. We asked faculty participants to locate their most recent curriculum vita before responding to questions about their publications and presentations.

We first asked participants how many peer-reviewed journal article publications in their career as a professor included undergraduate coauthors (not including undergraduate journals). For up to 20 publications with undergraduates (we thought 20 would be enough for most participants, and we also wanted to limit their time spent on the survey), we asked participants to list the year of publication, the number of authors (including themselves) and whether an undergraduate served as first author. Then, we asked faculty to estimate, in an average year, how many of their national or regional conference presentations included undergraduate coauthors and how many of those conference presentations typically included an undergraduate as first author.

### Analytic approach and reporting: Negative binomial regression

Given that both outcomes were count variables (i.e., number of publications or conference presentations), we used a form of regression specifically designed for count-type data: Negative binomial regression [37]. Negative binomial regression is a form of regression that neither assumes the normal distribution of errors—as linear regression does—nor equality of the mean and variance—as Poisson regression (another count-type regression) does.

We first examined bivariate relationships between each of our predictors and the four outcomes (publications with undergraduates, publications with undergraduates as first authors, presentations with undergraduates, presentations with undergraduates as first authors) by conducting a series of negative binomial regressions predicting each of the four outcomes from each predictor and an intercept. Then, as outlined in our preregistration, we examined these relationships controlling for institution type (i.e., PUI or GSI) and faculty members’ overall productivity (i.e., total number of publications). In doing so, we hoped to control for the variance associated both with the general expectations of a faculty member’s position (as a function of institution type) and of simply having greater research production. We believe the regression controlling for these variables to be a cleaner test of the factors that should influence research productivity with undergraduates.

After identifying factors that predicted our outcomes at the bivariate level, we conducted an exploratory analysis to examine the extent to which individual predictors best accounted for the data together. Specifically, we conducted a series of backward-elimination regressions to identify the relationships that explained the greatest amount of variance in our outcome parsimoniously. For each model (i.e., for each of the four primary dependent variables—publications, publications as first author, conference presentations, presentations as first author), we started with all predictors that emerged at the bivariate level and then systematically eliminated them by choosing the one with the weakest relationship until eliminating one significantly decreased model fit (i.e., we eliminated the weakest predictors one at a time until eliminating a predictor made the overall model significantly worse at predicting the outcome). The results from the final model from each backward-elimination stepwise approach appear in Table 4. These predictors represent the variables that, together, offered the most parsimonious prediction of each outcome, controlling for the other variables in the model.

Although model overfitting in backward elimination regression can be an issue, we chose to conduct these analyses for three reasons: First, in the broadest sense, this study is exploratory—our goal was for it to be the first step in a broader investigation of outcomes of undergraduate research in psychology. At the bivariate level, we had several a priori hypotheses. However, given the extremely limited literature and sheer number of exploratory variables in this study, we had no hypothesis-driven way of choosing predictors to pit against one another. Second, many of the predictor variables might represent constructs that are totally captured by other variables (e.g., the cost/benefit ratio of mentoring undergraduates might simply represent a combination of institutional support, enjoyment of mentoring undergraduates, and student quality). By pitting the variables against one another in this way, we were able to isolate the variables that best explained the outcome while eliminating redundant variables. Finally, these regression analyses offered a follow-up test to synthesize information learned at the bivariate level. In short, our goal was not to represent our results as the ultimate “truth” regarding predictors of undergraduate research outcomes; instead, we wanted to offer additional insight into the information we learned at the bivariate level. Thus, although we recognize that backward elimination regression is not the most ideal approach to understanding which predictors are strongest, we sought to provide additional exploratory information to help readers better understand the patterns we observed.

Prior to analysis, we z-scored all of the continuous and polytomous predictors so that the regression estimates would represent the change in outcome that accompanied a standard deviation change in the predictor. This adjustment allows for comparison of effect sizes for predictors that are on vastly different scales (e.g., 1–5 vs. 10s of millions). Dichotomous variables were coded as -.5 and .5, and thus the regression results represent a direct comparison between the two categories.

We report our results as incidence rate ratios (IRRs). IRRs represent the percentage change in the outcome one would expect for one unit of change in the predictor. Because we standardized (z-scored) the predictor variables prior to estimation, the IRRs represent the percentage change in the outcome expected alongside a one standard deviation increase in the continuous or polytomous predictor variables, or between the two categories of the dichotomous predictor variables. Incidence rate ratios are centered around 1.00, such that an incidence rate ratio of 1.00 suggests no difference between the two variables (i.e., at a one-step change in the predictor, one would expect the outcome to be 100% of what it was before—that is, unchanged). Numbers below 1.00 indicate a decline in the outcome with an increase in a predictor (e.g., at an incidence rate ratio of 0.80, one would expect that a one-step change in the predictor would correspond to the outcome being 80% of what it was before—a 20% reduction). Numbers above 1.00 indicate an increase in the outcome with an increase in a predictor (e.g., at an incidence rate ratio of 1.20, one would expect that a one-step change in the predictor would correspond to the outcome being 120% of what it was before—a 20% increase).

We present all results in Tables 2–4. Columns labeled “no controls” represent the simple bivariate relationship between the predictor variable and the relevant outcome (i.e., publications or presentations). Columns labeled “Controlling for PUI status and overall productivity” contain the results from a negative binomial regression examining the relationship between the focal predictor and the relevant outcome while controlling for institution type and total number of publications.

**Table 3:** Negative binomial regressions examining predictors of publications with undergraduate (UG) authors (left estimates) and publications with UG first authors (right estimates).

	# Publications with UGs		# Publications with UGs as First Author		
	No Controls	Controlling for PUI status and Productivity	No Controls	Controlling for PUI status and Productivity	
Variable	Standardized IRR [CI_95%_]^1^	Standardized IRR [CI_95%_]^1^	Standardized IRR [CI_95%_]^1^	Standardized IRR [CI_95%_]^1^	Interpretation
**Institution**					
PUI v Grad-Serving^2^	.92 [.70, 1.22]	**.65 [.48, .88]**	.73 [.50, 1.06]	**.63 [.43, .94]**	PUIs: 154% more pubs. with UGs; 159% more pubs. with UGs as first author
University Rank Within Type	1.04 [.89, 1.20]	.85 [.71, 1.03]	1.05 [.85, 1.31]	1.02 [.80, 1.29]	
Endowment	**1.30 [1.07, 1.58]**	1.13 [.93, 1.37]	1.06 [.87, 1.30]	.84 [.65, 1.08]	
Acceptance Rate^3^	**.76 [.64, .90]**	**.83 [.70, 1.00]**	.91 [.74, 1.12]	1.05 [.84, 1.32]	1 SD increase in acceptance rate: 17% fewer pubs. with UGs
Student-Faculty Ratio	**.83 [.70, .99]**	.80 [.63, 1.01]	.86 [.67, 1.09]	1.03 [.74, 1.42]	
Institutional Support for UG Research	1.11 [.97, 1.27]	1.15 [.99, 1.34]	**1.32 [1.10, 1.59]**	**1.27 [1.05, 1.55]**	1 SD increase in support for UG research: 27% more pubs. with UGs as first author
**Department**					
# of Faculty	1.11 [.95, 1.31]	1.14 [.95, 1.37]	1.02 [.86, 1.22]	1.09 [.90, 1.32]	
UG Research in Curriculum	1.08 [.95, 1.23]	**1.19 [1.01, 1.39]**	**1.34 [1.11, 1.63]**	**1.28 [1.04, 1.59]**	Departments with UG research in curriculum: 19% more pubs. with UGs; 28% more pubs. with UGs as first author
UG Research in Course Load	1.06 [.80, 1.40]	1.04 [.77, 1.41]	**1.53 [1.05, 2.22]**	1.41 [.95, 2.09]	
Course Load	**.78 [.68, .90]**	**.80 [.68, .95]**	**.73 [.58, .93]**	.79 [.62, 1.00]	1 SD increase in course load: 20% fewer pubs. with UGs
**Faculty Characteristics**					
Age	**1.57 [1.33, 1.84]**	1.16 [.96, 1.39]	1.52 [1.26, 1.82]	1.21 [.97, 1.51]	
Female v. Male	**1.43 [1.06, 1.92]**	.98 [.71, 1.36]	**1.53 [1.04, 2.25]**	1.08 [.71, 1.64]	
Years as a Full-Time Faculty member	**1.70 [1.45, 2.00]**	**1.24 [1.02, 1.51]**	**1.62 [1.35, 1.95]**	**1.33 [1.06, 1.67]**	1 SD increase in years as a full-time faculty member: 24% more pubs. with UGs, 33% more pubs. with UGs as first author
Tenure Track Rank	**2.21 [1.81, 2.71]**	**1.62 [1.29, 2.04]**	**1.87 [1.42, 2.46]**	**1.42 [1.05, 1.92]**	1 step increase in tenure track rank: 62% more pubs. with UGs, 42% more pubs. with UGs as first author
Grant Recipient (> $5k)	**1.80 [1.35, 2.40]**	**1.50 [1.06, 2.12]**	1.14 [.78, 1.67]	1.14 [.75, 1.74]	Grantees publish 50% more pubs. with UGs than do faculty without grants.
# of Publications	**2.29 [1.76, 2.99]**	**2.41 [1.85, 3.13]**	**1.64 [1.32, 2.03]**	**1.67 [1.34, 2.08]**	1 SD increase in productivity: 141% more pubs. with UGs 67% more pubs. with UGs as first author
Hours Worked Per Week	1.07 [.93, 1.22]	1.03 [.87, 1.23]	1.16 [.96, 1.39]	1.10 [.91, 1.34]	
Hours Spent on Research	**1.49 [1.29, 1.73]**	**1.33 [1.09, 1.61]**	**1.34 [1.10, 1.63]**	**1.26 [1.00, 1.59]**	I SD increase in hours spent on research: 33% more pubs. with UGs, 26% more pubs. with UGs as first author
Hours Spent Teaching Per Course	1.01 [.88, 1.15]	1.05 [.90, 1.23]	1.19 [.97, 1.46]	1.19 [.96, 1.47]	
Satisfaction With Current Position	1.14 [.99, 1.30]	**1.23 [1.06, 1.43]**	**1.52 [1.24, 1.86]**	**1.37 [1.11, 1.69]**	1 SD increase in satisfaction with their current position: 23% more pubs. with UGs, 37% more pubs. with UGs as first author
**Research Lab**					
# Grad Students in Lab^4^	**1.25 [1.00, 1.56]**	1.09 [.89, 1.32]	1.01 [.77, 1.32]	.95 [.72, 1.25]	
# Undergrads in Lab	1.19 [.99, 1.43]	1.12 [.93, 1.34]	**1.31 [1.11, 1.55]**	1.14 [.95, 1.37]	
Experience with Diverse Students	**1.27 [1.10, 1.45]**	1.07 [.92, 1.25]	1.15 [.96, 1.38]	1.05 [.87, 1.26]	
**Collaboration**					
% of RAs Who Are Juniors or Seniors	**.74 [.63, .87]**	.87 [.73, 1.04]	**.80 [.67, .96]**	.97 [.79, 1.19]	
% of RAs Who Are Primarily "Assistants"	.98 [.84, 1.13]	.97 [.82, 1.13]	**.77 [.63, .94]**	**.81 [.66, .99]**	1 SD increase in the % of UG research assistants that are “Assistants”: 19% fewer pubs. with UGs as first author
% of RAs Who Are Primarily "Collaborators"	1.00 [.87, 1.16]	1.07 [.91, 1.26]	1.00 [.83, 1.21]	1.18 [.95, 1.46]	
Length of Collaboration with Undergrads	**1.18 [1.03, 1.35]**	**1.20 [1.04, 1.40]**	.90 [.74, 1.08]	.97 [.80, 1.19]	1 SD increase in length of collaboration with undegraduates:18% more pubs. with UGs
**Collaborations with other Faculty**					
Primarily Students (No Outside Faculty)	.94 [.82, 1.08]	.97 [.83, 1.13]	1.02 [.84, 1.23]	1.03 [.85, 1.26]	
Within Department	1.00 [.87, 1.15]	.98 [.84, 1.15]	.94 [.77, 1.15]	.90 [.74, 1.11]	
Outside Dept, Within Univ.	1.03 [.90, 1.17]	.92 [.79, 1.08]	.86 [.71, 1.04]	.89 [.72, 1.09]	
Outside the University	**1.31 [1.13, 1.54]**	**1.18 [1.00, 1.38]**	.92 [.75, 1.12]	.99 [.81, 1.22]	1 SD increase collaboration with other faculty outside the university:18% more pubs. with UGs
**Collaborations with Students**					
Primarily Work Alone on Projects (No Students)	**.85 [.74, .98]**	**.83 [.71, .97]**	.96 [.79, 1.17]	.88 [.71, 1.08]	1 SD increase in working on projects alone: 17% fewer pubs. with UGs
Primarily Undergraduate Lab	.95 [.82, 1.09]	**1.19 [1.01, 1.40]**	**1.23 [1.02, 1.49]**	**1.38 [1.10, 1.73]**	1 SD increase in endorsement that their lab is “primarily undergraduate”: 19% more pubs. with UGs 37% more with UGs as first author
Both Grad and Undergrad Co-Investigators^4^	**1.46 [1.18, 1.81]**	1.13 [.90, 1.43]	1.25 [.93, 1.69]	1.13 [.83, 1.56]	
Primarily Grad, Expected Mentoring of UG^4^	1.22 [.98, 1.53]	1.10 [.86, 1.39]	1.01 [.75, 1.36]	.96 [.71, 1.29]	
PI Supervises Grad Who Supervises UG^4^	1.15 [.94, 1.41]	1.08 [.87, 1.33]	1.05 [.81, 1.35]	1.06 [.82, 1.37]	
**Faculty-Student Interaction Styles**					
Collegial Relationships with UGs	1.11 [.97, 1.28]	**1.18 [1.01, 1.37]**	**1.32 [1.07, 1.63]**	**1.35 [1.08, 1.68]**	1 SD increase in collegial interaction style: 18% more pubs. with UGs, 35% more with UGs as first author
UGs Impact Project Direction	1.02 [.89, 1.18]	1.00 [.86, 1.16]	**1.45 [1.20, 1.77]**	**1.39 [1.14, 1.70]**	1 SD increase in UGs significantly impacting project direction: 39% more pubs. with UGs as first author
Very Accessible to UGs	.95 [.82, 1.09]	1.01 [.87, 1.17]	1.21 [.99, 1.46]	**1.30 [1.05, 1.60]**	1 SD increase accessibility to UGs: 30% more pubs. with UGs as first author
Clear Expectations for UGs	**1.15 [1.01, 1.31]**	1.07 [.92, 1.24]	1.10 [.91, 1.33]	1.11 [.91, 1.35]	
**Project Characteristics**					
Original Projects (vs. Replications)	**1.27 [1.09, 1.48]**	**1.22 [1.04, 1.42]**	1.20 [.98, 1.47]	**1.24 [1.00, 1.54]**	1 SD increase in original projects: 22% more pubs. with UGs, 24% more pubs. with UGs as first author
Number of Projects Per Year	**1.19 [1.02, 1.39]**	1.14 [.97, 1.35]	1.15 [.97, 1.37]	1.12 [.94, 1.35]	
Study Length	**1.18 [1.01, 1.37]**	**1.19 [1.01, 1.39]**	1.16 [.98, 1.38]	1.16 [.98, 1.38]	1 SD increase in study length: 19% more pubs. with UGs
**Participants**					
Animals vs. Humans^5^	1.34 [.74, 2.44]	1.34 [.74, 2.44]	1.49 [.73, 3.03]	1.49 [.73, 3.03]	
Adults vs. Children^5^	**1.72 [1.07, 2.78]**	**1.72 [1.07, 2.78]**	1.15 [.63, 2.07]	1.15 [.63, 2.07]	Researchers whose primary participants are children: 72% more pubs. with UGs than those whose primary research participants are adults
Average # of Participants	.94 [.83, 1.06]	.97 [.84, 1.11]	.89 [.74, 1.07]	.94 [.78, 1.13]	
**Primary Data Collection**					
Online	**.54 [.40, .73]**	**.67 [.49, .93]**	**.55 [.37, .82]**	.75 [.50, 1.14]	Researchers whose primary data collection is online: 33% fewer pubs. with UGs
Individuals in Person	1.05 [.78, 1.41]	1.19 [.86, 1.64]	.86 [.58, 1.27]	.73 [.49, 1.10]	
Groups in Person	.95 [.70, 1.28]	.90 [.65, 1.26]	1.26 [.84, 1.88]	.97 [.63, 1.48]	
**Faculty Perceptions of Students & Research**					
Student Quality	**1.21 [1.07, 1.37]**	**1.22 [1.05, 1.41]**	**1.50 [1.23, 1.83]**	**1.43 [1.16, 1.76]**	1 SD increase in perceived student quality: 22% more pubs. with UGs, 43% with UGs as first author
Benefit (vs. Cost) of Research	1.04 [.91, 1.19]	1.07 [.92, 1.26]	**1.30 [1.08, 1.56]**	1.20 [.98, 1.45]	
Enjoyment of Mentoring UGs	**1.19 [1.04, 1.35]**	**1.28 [1.08, 1.51]**	**1.32 [1.07, 1.62]**	**1.33 [1.07, 1.65]**	1 SD increase in enjoyment of mentoring UGs: 28% more pubs. with UGs, 33% more pubs. with UGs as first author
Perceived Benefit to UG of conducting/presenting/publishing research (3-item index)	**1.22 [1.06, 1.41]**	**1.19 [1.03, 1.38]**	1.22 [.99, 1.50]	**1.33 [1.06, 1.67]**	1 SD increase in perceived benefits of research for undergraduates: 19% more pubs. with UGs, 33% more pubs. with UGs as first author

Notes

**Bolded estimates** indicate *p* < .05.

1 = IRR is incidence rate ratio, or the % of change in the DV one would expect between each unit of change in the predictor. IRRs of 1.00 indicate no change, IRRs above 1.00 indicate a positive relationship whereas IRRs below 1.00 indicate a negative relationship. The IRRs are standardized in that they are expressed in terms of change in the outcome per 1SD change in the predictor for polytomous and continuous variables. Dichotomous variables are coded as -.5, .5 so the outcomes are expressed as the difference between the two categories.

2 = The columns that control for PUI status and overall productivity only control for one of these variables when the other is the focal indicator.

3 = Higher scores indicates less selectivity.

4 = Graduate-serving institutions only.

5 = Appear in same model; entering both of these comparisons into a model simultaneously creates a comparison between animal researchers and human researchers and a contrast between researchers studying adults and children (ignoring animal researchers).

**Table 4:** Negative binomial regressions examining predictors of presentations with undergraduate (UG) authors (left estimates) and presentations with UG first authors (right estimates).

	# Publications with UGs		# Publications with UGs as First Author		
	No Controls	Controlling for PUI status and Productivity	No Controls	Controlling for PUI status and Productivity	
Variable	Standardized IRR [CI_95%_]^1^	Standardized IRR [CI_95%_]^1^	Standardized IRR [CI_95%_]^1^	Standardized IRR [CI_95%_]^1^	Interpretation
**Institution**					
PUI v Grad-Serving^2^	**1.49 [1.11, 2.00]**	**1.45 [1.06, 1.98]**	**1.54 [1.12, 2.12]**	**1.58 [1.13, 2.21]**	Grad serving institutions: 45% more presentations with UGS as authors; 58% more presentations with UGs as first author
University Rank Within Type	.97 [.81, 1.17]	.90 [.74, 1.10]	.93 [.77, 1.13]	.88 [.72, 1.09]	
Endowment	.85 [.71, 1.03]	.82 [.67, 1.00]	**.68 [.53, .87]**	**.67 [.52, .86]**	1 SD increase in endowment: 33% fewer presentations with UGs as first author
Acceptance Rate^3^	1.18 [.98, 1.41]	1.16 [.96, 1.40]	**1.40 [1.15, 1.72]**	**1.36 [1.10, 1.67]**	1 SD increase in acceptance rate: 36% fewer presentations with UGs as first author
Student-Faculty Ratio	1.17 [.97, 1.40]	1.07 [.85, 1.35]	1.21 [.99, 1.47]	1.09 [.86, 1.39]	
Institutional Support for UG Research	1.00 [.87, 1.16]	1.06 [.91, 1.23]	1.05 [.90, 1.22]	1.11 [.94, 1.31]	
**Department**					
# of Faculty	1.12 [.96, 1.31]	1.03 [.88, 1.21]	1.08 [.91, 1.28]	.93 [.77, 1.13]	
UG Research in Curriculum	.99 [.86, 1.15]	1.08 [.92, 1.28]	1.06 [.91, 1.25]	1.13 [.95, 1.35]	
UG Research in Course Load	.96 [.71, 1.29]	1.06 [.77, 1.44]	1.19 [.87, 1.64]	1.24 [.89, 1.74]	
Course Load	1.02 [.87, 1.20]	1.06 [.89, 1.27]	1.13 [.95, 1.34]	1.15 [.95, 1.39]	
**Faculty Characteristics**					
Age	.98 [.85, 1.12]	1.00 [.86, 1.17]	1.00 [.86, 1.16]	1.07 [.91, 1.26]	
Female v. Male	1.06 [.77, 1.45]	1.11 [.79, 1.56]	1.09 [.78, 1.53]	1.19 [.83, 1.69]	
Years as a Full-Time Faculty member	.97 [.84, 1.12]	1.00 [.85, 1.18]	.96 [.82, 1.12]	1.06 [.89, 1.27]	
Tenure Track Rank	.94 [.77, 1.14]	.97 [.78, 1.19]	.90 [.73, 1.10]	1.00 [.81, 1.25]	
Grant Recipient (> $5k)	1.26 [.93, 1.72]	1.17 [.82, 1.68]	1.05 [.76, 1.46]	1.09 [.74, 1.60]	
# of Publications	1.10 [.91, 1.31]	1.05 [.87, 1.26]	1.02 [.83, 1.24]	.96 [.78, 1.18]	
Hours Worked Per Week	1.02 [.89, 1.18]	.98 [.85, 1.14]	1.03 [.88, 1.21]	1.02 [.86, 1.21]	
Hours Spent on Research	**1.23 [1.06, 1.42]**	**1.21 [1.00, 1.46]**	**1.18 [1.00, 1.38]**	1.21 [.99, 1.49]	1 SD increase in hours spent on research each week: 21% more presentations with UGs annually.
Hours Spent Teaching Per Course	.90 [.78, 1.04]	.92 [.79, 1.07]	.88 [.75, 1.04]	.91 [.77, 1.08]	
Satisfaction with Current Position	.96 [.83, 1.11]	.96 [.83, 1.12]	.98 [.84, 1.14]	.98 [.83, 1.15]	
**Research Lab**					
# Grad Students in Lab^4^	1.18 [.95, 1.47]	1.17 [.93, 1.48]	1.13 [.91, 1.40]	1.17 [.92, 1.49]	
# Undergrads in Lab	**1.43 [1.19, 1.71]**	**1.36 [1.13, 1.63]**	**1.58 [1.29, 1.93]**	**1.53 [1.24, 1.87]**	1 SD increase in number of undergraduates in the labs: 36% more presentations with UGs, 53% more presentations with UGs as first author annually.
Experience with Diverse Students	**1.23 [1.05, 1.43]**	**1.19 [1.01, 1.40]**	1.18 [1.00, 1.40]	1.17 [.98, 1.40]	
**Collaboration**					
% of RAs Who Are Juniors or Seniors	.93 [.79, 1.09]	.93 [.78, 1.11]	.99 [.83, 1.18]	.90 [.75, 1.09]	
% of RAs Who Are Primarily "Assistants"	.92 [.79, 1.07]	.91 [.77, 1.07]	**.85 [.72, .99]**	**.78 [.66, .93]**	1 SD increase in % of undergraduates in the lab who are “assistants”: 22% fewer presentation with UGs as first author annually.
% of RAs Who Are Primarily "Collaborators"	**1.20 [1.03, 1.40]**	**1.22 [1.04, 1.43]**	**1.29 [1.09, 1.51]**	**1.37 [1.15, 1.63]**	1 SD increase in % of undergraduates in the lab who are “collaborators”: 22% more presentations with UGs, 37% more presentations with UGs as first author annually.
Length of Collaboration with Undergrads	**1.16 [1.00, 1.33]**	1.13 [.98, 1.32]	**1.18 [1.02, 1.36]**	1.16 [1.00, 1.35]	
**Collaborations with other Faculty**					
Primarily Students (No Outside Faculty)	1.02 [.88, 1.19]	1.01 [.86, 1.19]	1.12 [.95, 1.31]	1.09 [.92, 1.30]	
Within Department	1.06 [.91, 1.23]	1.02 [.87, 1.19]	1.06 [.91, 1.25]	1.03 [.87, 1.22]	
Outside Dept, Within Univ.	**1.15 [1.00, 1.33]**	1.13 [.97, 1.31]	1.13 [.97, 1.32]	1.09 [.92, 1.28]	
Outside the University	1.16 [.99, 1.36]	1.16 [.99, 1.36]	1.10 [.93, 1.30]	1.12 [.94, 1.33]	
**Collaborations with Students**					
Primarily Work Alone on Projects (No Students)	**.79 [.67, .93]**	**.79 [.66, .93]**	**.77 [.65, .92]**	**.77 [.64, .93]**	1 SD increase in endorsement that a faculty member works alone: 21% fewer presentations with UGs, 23% fewer presentations with UGs as first author annually.
Primarily Undergraduate Lab	1.05 [.91, 1.22]	1.19 [1.00, 1.41]	**1.24 [1.06, 1.45]**	**1.39 [1.16, 1.68]**	1 SD increase in endorsement that lab is primarily a UG lab: 39% more presentations with UGs as first author annually.
Both Grad and Undergrad Co-Investigators^4^	1.22 [.99, 1.52]	1.24 [.98, 1.56]	1.08 [.86, 1.35]	1.13 [.89, 1.45]	
Primarily Grad, Expected Mentoring of UG^4^	1.14 [.92, 1.42]	1.17 [.93, 1.47]	1.02 [.82, 1.27]	1.05 [.83, 1.34]	
PI Supervises Grad Who Supervises UG^4^	1.08 [.87, 1.33]	1.10 [.87, 1.39]	.98 [.79, 1.23]	.98 [.77, 1.26]	
**Faculty-Student Interaction Styles**					
Collegial Relationships with UGs	**1.20 [1.03, 1.40]**	**1.24 [1.06, 1.46]**	**1.41 [1.19, 1.67]**	**1.44 [1.21, 1.73]**	1 SD increase in collegiality with UGs: 24% more presentations with UGs, 44% more presentations with UGs as first author annually.
UGs Impact Project Direction	1.16 [1.00, 1.35]	**1.17 [1.01, 1.37]**	**1.33 [1.13, 1.56]**	**1.31 [1.11, 1.55]**	1 SD increase in allowing UGs to impact project direction: 17% more presentations with UGs, 31% more presentations with UGs as first author annually.
Very Accessible to UGs	1.01 [.87, 1.16]	1.05 [.90, 1.22]	1.12 [.96, 1.31]	1.13 [.96, 1.34]	
Clear Expectations for UGs	1.13 [.97, 1.31]	1.15 [.98, 1.35]	**1.19 [1.02, 1.39]**	**1.24 [1.05, 1.47]**	1 SD increase in setting clear expectations for UGs: 24% more presentations with UGs as first author annually.
**Project Characteristics**					
Original Projects (vs. Replications)	1.15 [.99, 1.32]	1.13 [.97, 1.31]	1.07 [.92, 1.25]	1.12 [.96, 1.32]	
Number of Projects Per Year	**1.42 [1.21, 1.67]**	**1.38 [1.16, 1.63]**	**1.52 [1.28, 1.80]**	**1.51 [1.26, 1.80]**	1 SD increase in # of projects annually: 38% more presentations with UGs, 51% more presentations with UGs as first author annually.
Study Length	1.12 [.97, 1.28]	1.09 [.94, 1.27]	1.10 [.95, 1.27]	1.06 [.91, 1.24]	
**Participants**					
Animals v. Humans^5^	.91 [.47, 1.74]	.91 [.47, 1.74]	.87 [.43, 1.77]	.87 [.43, 1.77]	
Adults v. Children^5^	.91 [.54, 1.53]	.91 [.54, 1.53]	.72 [.41, 1.28]	.72 [.41, 1.28]	
Average # of Participants	1.04 [.91, 1.20]	1.06 [.92, 1.23]	1.10 [.93, 1.30]	1.05 [.89, 1.24]	
**Primary Data Collection**					
Online	1.19 [.86, 1.63]	1.25 [.89, 1.76]	1.37 [.97, 1.94]	1.31 [.91, 1.89]	
Individuals in Person	1.36 [.98, 1.87]	1.32 [.95, 1.85]	1.37 [.97, 1.94]	1.35 [.94, 1.94]	
Groups in Person	.96 [.70, 1.32]	.99 [.71, 1.38]	.96 [.69, 1.36]	1.02 [.71, 1.46]	
**Faculty Perceptions of Students & Research**					
Student Quality	**1.28 [1.10, 1.48]**	**1.28 [1.10, 1.51]**	**1.32 [1.12, 1.55]**	**1.35 [1.14, 1.60]**	1 SD increase in perceived student quality: 28% more presentations with UGs, 35% more presentations with UGs as first author annually.
Benefit (vs. Cost) of Research	1.13 [.98, 1.30]	1.12 [.96, 1.31]	1.15 [.99, 1.34]	**1.19 [1.01, 1.40]**	1 SD increase in perceiving UG research as having more benefits than costs: 19% more presentations with UGs as first author annually.
Enjoyment of Mentoring UGs	**1.34 [1.14, 1.57]**	**1.34 [1.12, 1.59]**	**1.41 [1.17, 1.68]**	**1.42 [1.18, 1.72]**	1 SD increase in enjoyment of UG mentoring: 34% more presentations with UGs, 42% more presentations with UGs as first author annually.
Perceived Benefit to UG of conducting/presenting/publishing research (3-item index)	**1.25 [1.06, 1.48]**	**1.26 [1.06, 1.49]**	**1.33 [1.11, 1.60]**	**1.30 [1.08, 1.57]**	1 SD increase in perceived benefit of research to students: 26% more presentations with UGs, 30% more presentations with UGs as first author annually.

Notes

**Bolded estimates** indicate *p* < .05.

1 = IRR is incidence rate ratio, or the % of change in the DV one would expect between each unit of change in the predictor. IRRs of 1.00 indicate no change, IRRs above 1.00 indicate a positive relationship whereas IRRs below 1.00 indicate a negative relationship. The IRRs are standardized in that they are expressed in terms of change in the outcome per 1SD change in the predictor for polytomous and continuous variables. Dichotomous variables are coded as -.5, .5 so the outcomes are expressed as the difference between the two categories.

2 = The columns that control for PUI status and overall productivity only control for one of these variables when the other is the focal indicator.

3 = Higher scores indicate less selectivity.

4 = Graduate-serving institutions only.

5 = Appear in same model; entering both of these comparisons into a model simultaneously creates a comparison between animal researchers and human researchers and a contrast between researchers studying adults and children (ignoring animal researchers).

## Results

### Descriptive statistics

A description of faculty in the sample can be found in Table 1. Participants were more frequently female (68%) and predominantly White (92%). The average age was 46 and most faculty had tenure (81.2%). Respondents were also more likely to be from PUIs (49%) followed by master’s universities (26%) and doctoral universities (18%). On average, they had been at their current institution for 13.5 years and reported working approximately 50 hours per week. The average faculty course load was 5.5 courses per year, and they reported working with an average of 8.8 undergraduates on 3.8 projects per year. The average number of career publications was 21.8, and 35% had received at least one external grant over $5000 in the past 10 years.

### Predictors of publishing with undergraduates

Tables 3 and 4 present the results of four regression models. Table 3 presents the number of peer-reviewed publications with undergraduates, in general (left side of bolded line), and with undergraduates as first author (right side of bolded line). Table 4 presents the number of conference presentations with undergraduates, in general (left side of bolded line), and with undergraduates as first author (right side of bolded line).

The far-right column of the table offers a written interpretation of any significant findings for each predictor. Because we propose that the strongest test of these relationships is the one controlling for institution type and overall productivity, we report only those findings in the text. Nevertheless, we provide the estimates of all bivariate relationships for reader inspection in the table.

Faculty who reported their number of publications with undergraduates (*n* = 240) indicated an average of 5.28 publications coauthored with undergraduates (*Mdn* = 3; *SD* = 6.62). Faculty who reported authorship details of papers with undergraduates (*n* = 186) indicated an average of 1.55 publications with undergraduates as first author (*Mdn* = 1; *SD* = 2.65). One of the important questions this research sought to answer was whether institution type is related to publishing with undergraduates. As expected, after controlling for overall productivity, faculty at PUIs publish significantly more articles with undergraduates, both in general and with undergraduates as first author, than do faculty at graduate-serving institutions. In what follows we focus on significant findings as well as non-significant patterns of interest in the regression controlling for institution type and productivity. Comprehensive results for all predictors of publication with undergraduates can be found in Table 3.

#### Institutional variables

Faculty reported a greater number of publications with undergraduates to the extent that their institutions had lower acceptance rates (i.e., were more selective) and better support for undergraduate research, but neither of these variables predicted first-authored undergraduate publications. Contrary to predictions, university rank and endowment were unrelated to publishing with undergraduates.

#### Departmental variables

Faculty with higher course loads reported fewer publications with undergraduates in general, but not fewer publications with undergraduates as first author. Faculty in departments with undergraduate research scaffolded into the curriculum reported more publications with undergraduates as first author, but not more publications in general. Surprisingly, incorporating undergraduate research into the course load was unrelated to undergraduate publication (first author or otherwise).

#### Faculty characteristics

Faculty reported more publications with undergraduates, both in general and as first author, if they had more years of experience, were of higher tenure-track rank, had a greater number of publications, spent more hours on research, and were more satisfied with their current position. In addition, faculty who were grant recipients reported more publications with undergraduates but not more publications with undergraduates as first author. Gender, age, hours spent teaching per course, and total hours worked per week were not related to publishing with undergraduates (in general or as first author).

We also examined research area to determine whether it predicted undergraduate publication. In the interest of power, we restricted analyses to subfields with at least 20 faculty respondents, which included clinical/counseling (*n* = 29), cognitive (*n* = 42), developmental (*n* = 21), neuroscience (*n* = 22), and social (*n* = 75). A non-parametric independent-samples Kruskal-Wallis test (essentially a non-parametric one-way ANOVA) was marginally significant at the omnibus level for publications with undergraduates in general, *H*(4) = 9.28, *p* = .054, and significant at the omnibus level for publications with undergraduates as first author, *H*(4) = 12.24, *p* = .02. An examination of the pairwise comparisons suggested that faculty in neuroscience (*Md* = 5, *M* = 8.48, *SD* = 7.83) published with undergraduates significantly more than did faculty in developmental (*Md* = 2, *M =* 4.00, *SD* = 5.00; *H*(1) = 34.71, *p* = .03) and social psychology (*Md* = 2, *M =* 4.15, *SD* = 5.87; *H*(1) = 35.63, *p* = .007). Additionally, faculty in neuroscience (*Md* = 2, *M* = 3.00, *SD* = 3.01) published with undergraduate as first authors significantly more than did faculty in developmental (*Md* = 0.89, *M =* 1.00, *SD* = 1.36; *H*(1) = 36.57, *p* = .01), clinical/counseling (*Md* = 0, *M =* 1.12, *SD* = 1.59; *H*(1) = 30.55, *p* = .03), and social psychology (*Md* = 0, *M =* 1.05, *SD* = 2.39; *H*(1) = 40.15, *p* = .001). No other differences between areas were statistically significant.

#### Faculty perceptions of students and research

Faculty reported more publications with undergraduates, both in general and with undergraduates as first author, to the extent that they enjoyed mentoring undergraduates, believed research to be beneficial to undergraduates, and perceived their undergraduates as being higher-quality students. Surprisingly, perceiving the benefits of undergraduate research to outweigh the costs did not predict publishing with undergraduates.

#### Research lab variables

Faculty reported more publications with undergraduates, both in general and with undergraduates as first author, if they had a primarily undergraduate lab and reported establishing collegial relationships with undergraduates. Faculty also reported more publications with undergraduates, though no difference in undergraduate first authorship, to the extent that they did not work primarily alone on projects, had longer collaborations (e.g., a year vs. a semester) with undergraduates, or reported collaborative relationships with faculty outside the university (collaborative relationships within the department or university were not predictive).

Predictors of first-author undergraduate publication (but not undergraduate publication in general) included the degree to which faculty allowed undergraduates to significantly impact the direction of their research projects and made themselves accessible to undergraduates. Faculty also reported fewer publications with undergraduates as first author when their lab was comprised of research assistants who were primarily “assistants” rather than “collaborators.” No model of graduate lab (e.g., graduate students mentor and train undergraduates; graduate students and undergraduates collaborate as equals) predicted publishing rates with undergraduates, nor did the number of students (graduate or undergraduate) working in the lab.

#### Research project characteristics

Faculty who conducted more original (rather than replication) projects reported higher numbers of publications with undergraduates, both in general and with undergraduates as first author. Faculty also reported more undergraduate publications in general if their studies took longer to run, if they studied children rather than adults, and if they collected data primarily in person rather than online. Other forms of data collection, the comparison between human participants and animal subjects, and the number of projects with undergraduates completed each year did not relate to undergraduate publication (in general or as first author).

#### Identifying key predictors of publication

The top two sections of estimates in Table 5 present the final results of the backward elimination regression predicting publications with undergraduate coauthors in general and as first author. Three factors emerged as predictors of both types of publications: Faculty reported more publications with undergraduates in general and as first author when they were of higher tenure-track rank, spent more hours each week on research, and established close, collegial relationships with undergraduates. In addition to these three factors, faculty reported more publications with undergraduates in general to the extent that they had higher overall productivity (i.e., more total publications), had a primarily undergraduate lab, collaborated with faculty outside the university, and were at more selective institutions. Faculty also reported more publications with undergraduates as first author when they reported higher perceived student quality and allowed their undergraduates to influence the direction of their own research projects.

**Table 5:** Negative binomial regressions from the final model in a series of backward-elimination stepwise regressions.

Publications	Standardized IRR [CI_95%_]^1^	Interpretation
Tenure Track Rank	**1.95 [1.46, 2.61]**	1 step increase in tenure track rank: 95% more publications with undergraduates
Productivity	**1.39 [1.03, 1.89]**	1SD Increase in productivity: 39% more publications with undergraduates
Hours spent on research each week	**1.37 [1.04, 1.80]**	1SD Increase in hours spent on research each week: 37% more publications with undergraduates
Primarily Undergraduate Lab	**1.32 [1.07, 1.63]**	1SD Increase in agreement that one has a predominately undergraduate lab: 32% more publications with undergraduates
Collaboration with faculty outside the university	**1.29 [1.04, 1.60]**	1SD Increase in collaboration with other universities: 29% more publications with undergraduates
Collegial Relationships with UGs	**1.24 [1.01, 1.53]**	1SD Increase in close, collegial relationships with undergraduates: 24% more publications with undergraduates
Acceptance Rate	**.80 [.66, .98]**	1SD decrease in school selectivity: 20% fewer publications with undergraduates
Study Length	1.13 [.95, 1.35]	
**First-Authored Publications**		
Tenure Track Rank	**1.78 [1.33, 2.37]**	1 step increase in tenure track rank: 78% more publications with undergraduates as first author
Hours spent on research each week	**1.49 [1.19, 1.86]**	1SD Increase in hours spent on research: 49% more publications with undergraduates as first author
Perceived Student Quality	**1.31 [1.05, 1.63]**	1SD Increase in perceptions of student quality: 31% more publications with undergraduates as first author
UGs Impact Project Direction	**1.25 [1.00, 1.56]**	1SD Increase in undergraduate input on project direction: 25% more publications with undergraduates as first author
Collegial Relationships with UGs	1.23 [.97, 1.57]	
**Presentations**		
Enjoyment of Mentoring UGs	**1.26 [1.07, 1.49]**	1SD increase in enjoyment of mentoring undergraduates: 26% more presentations with undergraduate co-authors
Number of Projects Per Year	**1.25 [1.04, 1.50]**	1SD increase in number of projects undergraduates are involved in each year: 25% more presentations with undergraduate co-authors
Number of Undergraduates Per Year	**1.22 [1.00, 1.48]**	1SD increase in number of undergraduates in the lab each year: 22% more presentations with undergraduate co-authors
**First Author Presentations**		
Number of Projects Per Year	**1.57 [1.27, 1.95]**	1SD increase in number of projects undergraduates are involved in each year: 57% more presentations with undergraduates as first author
Collegial Relationships with UGs	**1.39 [1.10, 1.76]**	1SD increase in close, collegial relationships with undergraduates: 39% more presentations with undergraduates as first author
Endowment	**.71 [.56, .91]**	1SD increase in school endowment: 29% fewer presentations with undergraduates as first author

Notes: **Bolded estimates** indicate *p* < .05.

1 = IRR is incidence rate ratio, or the % of change in the DV one would expect between each unit of change in the predictor.

### Predictors of presenting with undergraduates

Faculty who reported their number of annual presentations with undergraduates (*n* = 243) indicated an average of 2.80 presentations with undergraduate coauthors per year (*Mdn* = 2; *SD* = 3.21). Faculty who reported conference presentations with undergraduates as first author (*n* = 237) indicated an average of 1.91 presentations with undergraduates as first author per year (*Mdn* = 1; *SD* = 2.92). In what follows we focus on significant findings as well as non-significant patterns of interest in the regression controlling for institution type and publication productivity. Comprehensive results for all predictors of conference presentations with undergraduates can be found in Table 3.

#### Institutional and departmental variables

Controlling for productivity, faculty at graduate-serving institutions reported significantly more presentations with undergraduate authors and undergraduate first authors than did faculty at primarily undergraduate institutions. Interestingly, faculty reported a greater number of presentations with undergraduates as first author to the extent that that their institutions had higher acceptance rates (i.e., they were less selective) and lower endowments. In addition, university rank and institutional support for undergraduate research were unrelated to presentations with undergraduate coauthors. No department variables were related to the number of presentations with undergraduate coauthors.

#### Faculty characteristics and perceptions

The only faculty characteristic that predicted presentations with undergraduate coauthors was hours spent on research: Faculty who spent more hours on research reported significantly more presentations with undergraduate co-authors. Unlike with publications, area of psychology did not predict conference presentations with undergraduates in general, *H*(4) = 2.00, *p* = .73, nor with undergraduates as first author, *H*(4) = 2.87, *p* = .58. Faculty perceptions of students and research also predicted presentations. Specifically, faculty reported more presentations with undergraduates, both in general and with undergraduates as first author, to the degree that they enjoyed mentoring undergraduates, believed research to be beneficial to undergraduates, and perceived their undergraduates as being higher-quality students. Moreover, faculty reported more presentations with undergraduate first authors (but not in general) when they perceived the benefits of mentoring undergraduate research to outweigh the costs.

#### Research lab and project characteristics

Faculty reported more presentations with undergraduate coauthors and first authors if they had a greater number of undergraduates in their lab, considered a greater percentage of undergraduate research assistants to be “collaborators” (rather than “assistants”), reported establishing collegial relationships with undergraduates, and allowed undergraduates to significantly impact the direction of their research projects. Not surprisingly, faculty reported fewer presentations with undergraduate coauthors and first authors if they worked primarily alone on projects. One additional factor predicted coauthored presentations with undergraduates in general, but not as first-author: Faculty who had greater experience with diverse students reported more presentations with undergraduate coauthors.

Predictors of first author undergraduate presentations (but not undergraduate presentations in general) included faculty reporting having primarily undergraduate labs and setting clear expectations for undergraduates. By contrast, faculty reported fewer presentations with undergraduates as first author to the extent that their lab was comprised of research assistants who were primarily “assistants” rather than “collaborators.” No model of graduate lab predicted presenting with undergraduates.

The only project characteristic that predicted undergraduate presentations was number of projects each year. That is, faculty had more conference presentations with undergraduate coauthors and undergraduate first if they conducted more projects each year.

#### Identifying key predictors of presentation

The bottom two sections of estimates in Table 4 present the final results of the backward elimination regression predicting presentations with undergraduate coauthors in general and as first author. Number of projects per year emerged as a predictor of both outcomes—faculty who conducted more projects with undergraduates each year also had more coauthored presentations with them, both in general and as first author. Similarly, faculty who reported having more undergraduates in their lab each year listed more coauthored presentations in general. Faculty who enjoyed mentoring undergraduates also reported more presentations with undergraduate coauthors in general. Furthermore, faculty reported more undergraduate first-authored presentations to the extent that they formed close, collegial relationships with undergraduates. Finally, faculty at schools with larger endowments reported fewer undergraduate first-authored presentations.

## Discussion

To the best of our knowledge, only two studies have examined predictors of undergraduate publication and presentation. One study was restricted to faculty from research-intensive institutions (i.e., biomedical faculty at 13 R1 institutions) and examined predictors of publication only [14], whereas the other was restricted to faculty from primarily undergraduate institutions (i.e., chemistry and physics faculty at 4 PUIs) and examined predictors of publication and conference presentation [13]. We sought to significantly extend the literature in this area by (a) including multiple institution types (research intensive/doctoral universities, master’s universities, and PUIs) in the same study to allow for a comparison of undergraduate-only and graduate-serving institutions, (b) including a larger number of individual institutions (*n =* 154; 52 participants did not specify their institution), (c) including a wider range of potential predictor variables, such as institutional variables (e.g., student selectivity, support for faculty-undergraduate research), which have been neglected in prior research, as well as research lab and project characteristics (e.g., typical participant, length of study, original vs. replication research), and (d) examining predictors of publication and conference presentation in which undergraduates serve as first author. We acknowledge that our study has limitations (as discussed below); nonetheless, several interesting patterns of results emerged that, with replication and cross-validation in future research, tell a “story” about the factors that lead to successful undergraduate research outcomes in psychology, and potentially other fields as well.

### The “best” predictors of undergraduate publication and presentation

Taken together, both the bivariate relationships and the results from the exploratory backward-elimination regression analyses shed light on key predictors of publication and presentation, at least in the context of the set of institutional, faculty, and project-related variables included in the present study.

#### Institution type

One of the primary goals of this research was to examine whether institution type is a factor in predicting undergraduate research outcomes. As expected, our results showed that, controlling for overall productivity, faculty at undergraduate-serving institutions (i.e., PUIs) publish more with undergraduates (both in general and as first author) than do faculty at graduate-serving institutions (GSIs). By contrast, faculty at GSIs (i.e., doctoral and master’s universities) coauthor more conference presentations (both in general and as first author) than do faculty at PUIs. These findings are consistent with Eagan et al. [32], who argued that faculty at PUIs (relative to those at GSIs) are more likely to include undergraduates in their research because the institutions are primarily focused on undergraduate education, small class sizes foster deeper connections with undergraduates, and because faculty are more likely to see collaborative research as mutually beneficial. In terms of student publication, faculty at GSIs generally prioritize graduate student research. They also may be supervising a large number of undergraduate research assistants at any one point in time, making it more likely that collaborative research with undergraduates would end in conference presentations, which require a lower investment and also leaves more publication author slots available for graduate students. At PUIs, the more intensive undergraduate focus [14], along with the lack of graduate students, makes it potentially more likely that collaborative faculty-undergraduate research would end in publication.

#### Faculty characteristics

An examination of Table 4 reveals that faculty characteristics appear to be more important than institutional characteristics in predicting undergraduate publication and presentation. Of the faculty characteristics, tenure track rank [(hich likely reflects faculty experience and skill in conducting research, with and without undergraduates [14, 21]) is the strongest predictor of publishing with undergraduates, both in general and as first author; that is, faculty of higher rank coauthor more publications with their students. Interestingly, faculty rank is not a factor in undergraduate conference presentations; it seems plausible that because the vast majority of research projects can lead to a conference presentation at a minimum, it is easier for even early-stage faculty to produce conference presentations with their undergraduate students, whereas publication would require greater effort and skill. As others have argued [33], it takes time and experience to learn how to mentor undergraduates, which is why later stage faculty may be more successful in publishing with undergraduates. Another potential reason faculty of higher rank have more publications (but not more conference presentations) with undergraduates is that untenured faculty may be concerned that the number of authors and/or the authorship order of publications could impact their tenure decision; by contrast, faculty who have already been tenured may feel more freedom to pursue and promote undergraduate authorship without worrying that doing so could harm their chances for professional advancement.

Hours spent on research was the second strongest predictor of publishing with undergraduates (in general and as first author). This finding indicates that, in addition to experience, skill, and practice mentoring undergraduates, faculty need time to conduct their research with undergraduates. The fact that course load was negatively related to publication at the bivariate level (i.e., faculty who taught fewer courses per year were more likely to publish with undergraduates) also suggests the importance of time to facilitate faculty-undergraduate publication.

Who faculty choose to collaborate with also matters. Not surprisingly, faculty who run a primarily undergraduate lab (more common at PUIs than GSIs) are more likely to publish with undergraduates, both in general and as first author. Faculty who publish with undergraduates also appear to benefit from the support and resources they get from collaborating with colleagues at other universities, which not only provides additional collaborators to generate ideas, conduct studies, and analyze data, but also access to more expensive equipment or tools that may not be available at a faculty member’s own university ([38, 39], as suggested by [40]). The latter interpretation is consistent with our finding that collaborating with faculty in the department or university did not predict undergraduate publication but collaborating with faculty at other universities did. Taken together with the observed relationship between grant funding and publication (see also [12]), our findings suggest that resources—monetary and otherwise—are important in facilitating undergraduate publication.

As expected, for both publishing and presenting with undergraduates, faculty mentoring style emerged as important, even in the context of all other predictors. Specifically, we found that (a) having close collaborative relationships predicted number of publications and first author presentations, (b) allowing undergraduates to impact project direction predicted first-author undergraduate publications, and (c) enjoyment of, and commitment to, mentoring undergraduates predicted number of publications, first author publications, and coauthored conference presentations (consistent with [14]). Taken together, these findings suggest a profile of the productive faculty mentor of undergraduates as someone who is undergraduate focused in their orientation and nurtures intensive, close collaborations with them, which is more likely to occur at PUIs (but which is obviously still possible at graduate serving universities).

#### Student quality and training

As predicted, and consistent with past research on engaging undergraduates in research in general [15, 21, 22], our results indicated that having access to high-quality students plays a role in publishing with undergraduate coauthors. Specifically, we found that more selective schools produce higher numbers of undergraduate publications, and that faculty who perceive that their institution has high quality, well-trained students are more likely to publish with undergraduates as first author. By contrast, for presenting (but not publishing) with undergraduates, sheer numbers are more critical, reflecting quantity over quality: Faculty who work with a larger number of undergraduates are more likely to coauthor presentations with them, and faculty who conduct more projects per year are also more likely to coauthor presentations.

### Limitations

The primary limitation of the current study is the non-representative nature of our sample. Because we used convenience and snowball sampling to attain a larger sample, we have overrepresented White, female, older, and tenured faculty. Faculty in our sample were also slightly more likely to be from PUIs than GSIs and in the area of social psychology relative to other subfields (two of the authors are social psychologists, which could explain the overrepresentation of this subfield). Perhaps more importantly, there is almost certainly a selection bias given that we actively recruited faculty who had prior experience working with undergraduates; moreover, volunteers who responded to a survey about undergraduate publication not only have more interest in and experience with the issue, but also more success in publishing with undergraduates relative to faculty who did not participate. This selection bias potentially restricted the range of some of our variables—especially those relating to faculty perceptions—which could lead to an underestimation of some important effect sizes. Although our results shed light on several important predictors of undergraduate publishing and presentation, they clearly need to be replicated in a more representative, diverse sample to ensure generalizability.

Next, the sample size was relatively small given the number of predictor variables tested. Although we attained as large of a sample as possible, asking faculty to respond to a survey at the beginning of global pandemic led to low response rates. Because there is so little empirical research on this topic and we wanted to maximize efficiency in this new research area by exploring as many variables as possible, there was not sufficient power nor variance to test interactions between variables, such as institution type and other predictor variables. Relatedly, the large number of tests conducted here leads to sizable familywise Type-1 error inflation—it is very likely that one or more of the observed relationships here emerged simply by chance. As such, we recommend that researchers interested in particular predictors replicate the present work in their domain of interest.

In addition, although we make directional inferences from our findings based on a priori hypotheses about various predictors and outcomes, reverse causality is always possible with any correlational study. As one example, we argue that faculty who dedicate more time to their research publish more frequently with undergraduates. It is also possible that faculty who publish more frequently with undergraduates (particularly those who enjoy mentoring undergraduates) are willing to invest more total time on research in order to also publish with undergraduates.

Finally, in what is perhaps more of a caveat than a limitation, the current study’s focus on the outcomes (i.e., publication and presentation) of faculty-undergraduate research collaboration should not be taken to mean that only research that produces these outcomes is important or beneficial. As noted earlier, there is a large body of research documenting the numerous benefits of such collaborations ([e.g., 41, 42]) and there are a wide range of possible models that successfully engage undergraduates in research. Nonetheless, because the dissemination of research findings is both a crucial component of the research process [13] as well as helpful for graduate school admission [4], we hope that by identifying factors that predict undergraduate research outcomes, the current study might help institutions to improve the quality and success of their undergraduate research programs.

### Suggestions for future research

Despite its limitations, our study suggests several promising avenues for future research. For example, we measured faculty research area to examine whether there were differences between subfields in publishing with undergraduates. Interestingly, we found that faculty in neuroscience were more likely to publish with undergraduates in general than were faculty in developmental or social psychology, and more likely to publish with undergraduates as first author than were faculty in developmental, social, or clinical psychology. One potential explanation for this finding is that neuroscience (compared to other subfields in psychology) is more similar to STEM fields, which tend to conduct their undergraduate research within organized, structural frameworks and consequently lead to more undergraduate engagement than do non-STEM fields [19]. Because these analyses were exploratory, consisted of relatively small sample sizes, and only five subfields were represented, they should be interpreted with caution and considered a starting point. Nonetheless, it would be fruitful for future research to replicate these findings with a larger sample and variety of subfields to determine if there are differences related to undergraduate publication and if so, whether these differences reflect trainable factors (e.g., aspects of the faculty-undergraduate mentoring relationships or organizational structure of the research program) or are merely artifacts of the publishing culture in that subfield (e.g., greater number of authors on a publication or higher number of publications overall).

Another focus for future research would be to more closely examine the process by which faculty successfully publish with undergraduates at graduate-serving institutions. We have seen (both anecdotally and in the current sample) that many faculty at graduate-serving institutions publish regularly with undergraduates, but the mechanisms for that process are to date unclear. In our study, neither the number of graduate students (consistent with [14]) nor the level of involvement and mentoring by graduate students was related to publishing with undergraduates, despite indirect evidence [43, 44] and theoretical arguments [45, 46] to the contrary. Further research in this area would allow us to determine the extent to which a successful model of publishing with undergraduates at GSIs is similar to—or completely different from—the model of publishing with undergraduates at PUIs.

We attempted to include a wide variety of project-related variables in our study because with few exceptions, such variables have been unexamined in prior research on undergraduate publication. We found that faculty who work with students for a longer period of time (e.g., a year or more) are more likely to publish with undergraduates (consistent with 14), which makes sense given the time it takes for a project to result in a published article. The fact that undergraduate publications (including first-authored publications) are more likely for original research makes sense given that original research tends to be easier to publish in general. Replication studies can take multiple years and are increasingly likely to be multi-site efforts. Moreover, even though publication of replications is becoming more common, it might still take several years for faculty publication records with undergraduates to reflect this trend. Future research replicating and extending our findings related to project characteristics, as well as studies that examine possible mechanisms, could provide a more complete picture and broaden our understanding of how project characteristics might influence publishing with undergraduates.

Finally, although our survey was fairly extensive, there are several additional questions that, in hindsight, we wish we had examined. For example, does a faculty member’s own undergraduate institution (e.g., its rank and/or type of institution) predict their mentoring style and likelihood of publishing and presenting with undergraduates? Similarly, to what extent does a faculty member’s own undergraduate research experience (e.g., whether they coauthored publications or presentations) influence the likelihood that they publish and present with undergraduates? We also did not address the timeline for publishing with undergraduates. It is almost certainly the case that publishing articles with undergraduates (especially with undergraduates as first author) takes longer than does publishing solo or with faculty or graduate student coauthors. As such, it would be interesting to explore in future research the existence of time lag differences (between publishing with and without undergraduate coauthors) to determine how long it takes for undergraduate-coauthored publications to appear on faculty vitae as well as the extent to which such time lag differences could help explain the relationship between faculty rank and increased publication (e.g., because Associate and especially Full Professors may feel less time pressure to publish quickly). In addition, although we measured faculty race, the lack of diversity in our sample meant that we were unable to examine whether race is related to faculty-undergraduate publication and presentation. Given research showing that the positive effects of faculty-undergraduate collaboration are even stronger for underrepresented groups [47–49], that faculty with prior experience mentoring African-American students are more likely to publish with undergraduates [14], and that in some cases, the match between student and faculty race is a factor in undergraduate publication [13], race should be a focus for future research to improve our understanding of the broader context in which undergraduate publication occurs.

## Conclusion and implications

Publishing and presenting with undergraduates seems heavily dependent on the faculty mentor. The fact that faculty of higher rank are more successful at publishing with undergraduates (yet are no more likely to coauthor presentations) suggests that faculty training on mentoring undergraduate research earlier in their career may be useful (see [46]). That is, it seems plausible that conference presentations are more of an endpoint for less experienced faculty (as well as for faculty at GSIs, who have graduate students that need authorships to make them competitive job candidates), whereas more experienced faculty (as well as those at PUIs, who have no graduate students competing for authorships) might use conference presentations as a steppingstone on the path to publication.

In addition, although institutional factors may not play as large of a role in undergraduate publication as do faculty factors, institutions can—and should—do more to encourage and support faculty in their research with undergraduates. In our study, faculty who reported that their administration encourages, supports, and rewards conducting and publishing research with undergraduates were more likely to publish with them. Engaging undergraduates in publishable research seems to be, at least in part, a resource issue (i.e., requiring time and funding), and it is critical to remove the barriers that prevent faculty from conducting publishable research with undergraduates. For example, institutions can adopt policies to encourage and reward faculty for undergraduate publication (as suggested by [4]), they can provide faculty time (e.g., in the form of reduced course loads or the incorporation of undergraduate research into the course load, as suggested by [7]), they can provide money and resources (e.g., summer funding and stipends for students and faculty), and they can encourage and facilitate cross-collaboration with faculty at other institutions. Such institutional changes have the potential to not only help faculty who already collaborate with students transition into coauthoring with them but could also encourage faculty who have yet to mentor undergraduates to begin engaging in faculty-undergraduate research. Indeed, a recent study found that a vast majority (80%) of faculty surveyed who had not mentored undergraduate research in the past said they would do so if such barriers were removed [6].

In closing, the current study represents an important initial step in establishing our understanding of the extensive factors that play a role in undergraduate publication and presentation in psychology. Simply put, our results suggest that the key characteristics of faculty who publish and present with their undergraduates are that they enjoy mentoring undergraduates and develop close, collegial relationships with them. In addition, whereas sheer numbers (of students to work with) seem to predict undergraduate presentations, to actually publish with undergraduates, faculty need experience, time, resources, and high-quality students. We hope that our study not only stimulates further research on this increasingly popular and important topic, but ultimately, that it encourages more faculty to see publication with undergraduates as an achievable goal, thus pushing “high impact” research experiences to even greater heights.
